# On the Use of a Molten Salt Fast Reactor to Apply an Idealized Transmutation Scenario for the Nuclear Phase Out

**DOI:** 10.1371/journal.pone.0092776

**Published:** 2014-04-01

**Authors:** Bruno Merk, Ulrich Rohde, Varvara Glivici-Cotruţă, Dzianis Litskevich, Susanne Scholl

**Affiliations:** Department of Reactor Safety, Institute of Resource Ecology, Helmholtz-Zentrum Dresden-Rossendorf, Dresden, Germany; Karlsruhe Institute of Technology (KIT), Germany

## Abstract

In the view of transmutation of transuranium (TRU) elements, molten salt fast reactors (MSFRs) offer certain advantages compared to solid fuelled reactor types like sodium cooled fast reactors (SFRs). In the first part these advantages are discussed in comparison with the SFR technology, and the research challenges are analyzed. In the second part cycle studies for the MSFR are given for different configurations – a core with U-238 fertile, a fertile free core, and a core with Th-232 as fertile material. For all cases, the transmutation potential is determined and efficient transmutation performance for the case with thorium as a fertile material as well as for the fertile free case is demonstrated and the individual advantages are discussed. The time evolution of different important isotopes is analyzed. In the third part a strategy for the optimization of the transmutation efficiency is developed. The final aim is dictated by the phase out decision of the German government, which requests to put the focus on the determination of the maximal transmutation efficiency and on an as much as possible reduced leftover of transuranium elements at the end of the reactor life. This minimal leftover is achieved by a two step procedure of a first transmuter operation phase followed by a second deep burning phase. There the U-233, which is bred in the blanket of the core consisting of thorium containing salt, is used as feed. It is demonstrated, that transmutation rates up to more than 90% can be achieved for all transuranium isotopes, while the production of undesired high elements like californium is very limited. Additionally, the adaptations needed for the simulation of a MSFR, and the used tool HELIOS 1.10 is described.

## Introduction

In the today’s view molten salt reactors have a long history, based on a, somewhat curious idea of the early phase of nuclear development in the late 40s and early 50s. >>The idea of using molten fluoride salts and thus liquid nuclear fuel in a reactor is rather old. Molten salt reactors were already proposed during the post-World War II attempt to design the nuclear powered aircraft. The Aircraft Reactor Experiment, a small thermal reactor (2.5MW) using circulating molten salt, operated for several days in 1953<< [1]. This first experiment has been followed by a larger scale experiment with 8 MW thermal, the Molten Salt Reactor Experiment (MSRE).>>Design of the MSRE started in the summer of 1960 and construction started 18 month later, at the beginning of 1962. The reactor went critical in June 1965, and was briefly at full power a year later << [1]. A major step in the MSRE was the demonstration of the use of thorium as fertile material and U-233 as fissile material. The reactor was operated until December 1969 and the U-235 fuel salt was successively replaced with U-233. Finally, the reactor was operated with U-233 fuel for several months. It was the first time U-233 has been used as reactor fuel. [1]

The molten salt reactor technology has attracted some new interest which was focused in the EURATOM project MOST – review on MOlten Salt reactor Technology [2], [3]. Following the MOST project, two recent important projects have been launched EVOL [4], [5] and MOSART [6], [7]. This new interest can be explained by some really interesting features of the molten fluoride salts. >>Molten fluoride salts have some beneficial characteristics, like the wide range of solubility of uranium and thorium, the thermodynamic stability and the resistance against radiologic decomposition, the low vapor pressure at operation temperature and the compatibility with nickel based alloys which are traditionally used as construction material<< [1]. In contrast to the MSRE, both current projects focus on the development of a molten salt reactor with fast neutron spectrum, thus without graphite used for moderation in the MSRE.

In addition to the specific features of the fluoride salts, molten salt fast reactors (MSFRs) exhibit large negative temperature and void reactivity effects. These large negative effects lead to a favorable, much more stable operational behavior than in classical fast reactors with solid fuel. The strong negative feedback effects are a unique safety characteristic which is not found in any kind of solid-fuelled fast reactors [8] and lead consequently to superior inherent safety characteristics. MSFR systems have been recognized as a long term alternative to solid-fuelled fast-neutron systems due to several additional unique favorable features (smaller fissile inventory, easy in-service inspection, simplified fuel cycle, etc.) [9].

In the frame of the development of future energy resources and an improved nuclear waste management, the specific molten salt reactor concept offers a large capability of different operational regimes. Previous studies led to the definition of the MSFR concept, which is now one of the six concepts selected by the Generation IV International Forum (GIF) [9] for the further study. In contrast to molten salt reactors previously studied, the specificity of the MSFR is the removal of any solid moderator (usually graphite) from the core. This choice is motivated by the study of parameters such as feedback effects, breeding ratio, graphite lifespan, and U-233 initial inventory [8]. This change results in a fast neutron spectrum molten salt reactor [10]. The MSFR is proposed to be operated in the Th/U-233 fuel cycle with fluoride salts. Since U-233 does not exist in nature, it is foreseen to start the reactor based on plutonium and minor actinides (TRUs or transuranium isotopes), produced in currently operating reactors as fissile material [11].

This study is focused on the optimization of the transmutation performance in the case of a phase out of energy production in nuclear power plants, like it is envisaged in Germany.

As a result of the Fukushima event, the German government has decided to close down 8 nuclear power plants (NPPs) immediately. The remaining 9 NPPs will be staggered shut down, with the last 2 ones in 2022 [18]. Former studies on the transuranium isotope (TRU) accumulation due to the operation of the NPPs lead to the following approximate TRU amounts in Germany at the end of the nuclear reactor operation period: ∼135 t plutonium, ∼13.5 t americium, and ∼1.7 t curium [14], [15], [16]. These amounts of TRU were foreseen to be put to a final repository when/if a location is found. The absolute amounts are slightly reduced due to the rapid close down of 8 plants. The most recent studies considering the nuclear phase out decision predict 131 t plutonium, 6.2 t neptunium, 14.6 t americium, and 0.7 t curium [17] in 2022. In addition to the final disposal in deep geologic formations the waste management strategy of Partitioning and Transmutation (P&T) is under intensive discussion in Germany now [19].

The major questions are: Do MSFRs show significant advantages for TRU transmutation in the case of a nuclear phase out? Which MSFR configuration offers the most efficient TRU transmutation performance? Is it possible to solve the so-called last transmuter problem efficiently in an MSFR by burning more than 90% of the TRUs?

P&T is recognized as an alternative to the direct final disposal of burnt reactor fuel. But with the special view of the nuclear phase out, there is always the limitation of the remaining last reactor load – the so-called last transmuter problem. Thus up to now the transmutation is foreseen mostly as an option in combination with continued operation of the nuclear reactor park. The reason for this is that transmutation is investigated and evaluated mostly on the basis of GEN-IV requirements: >>Generation IV nuclear energy systems will minimize and manage their nuclear waste and notably reduce the long-term stewardship burden, thereby improving protection for the public health and the environment<< [20]. Consequently, the option P&T with the focus on the special problems arising in conjunction with the phase out of electric energy production in nuclear power plants in Germany is not studied in the frame of the GEN-IV initiative at all.

## MSFR Features for Transmutation

A reactor with fast neutron spectrum is essential for an efficient transmutation of transuranium isotopes. A wide range of experience in fast reactor design, construction, and operation is available only for sodium cooled fast reactors (SFRs). The existing sodium cooled fast reactors have shown excellent operational behaviour within the last 10 years, and new designs and methods are available to improve safety significantly. This feature makes SFRs the most mature choice in fast reactor technology [24] today. Additionally, the technical feasibility of the transmutation of plutonium and minor actinides has been demonstrated in the PHÉNIX reactor [25], a SFR. Nevertheless, it has to be recognized that the early SFRs, designed in the 1970s, have been designed with the objective of efficient breeding of new fissile materials and not for the transmutation of TRUs.

The new SFR designs, which are currently under development, follow other design criteria than those used for systems dating from the 1970s. Fuel breeding is not the major task anymore; thus, well-balanced systems are designed with optimized feedback effects, which results in excellent safety behavior [21]. Additionally, the recently investigated use of fine distributed moderating material offers the possibility of designable feedback effects, since the most important inherent feedback effects, the Doppler and the coolant effect, can be influenced significantly without negative implications to the other safety and operationally relevant system parameters [26], [27], [28], [29]. Unfortunately, the amount of TRUs in the core of a SFR is strictly limited due to the negative influence of some TRU isotopes on the inherent safety effects of the reactor core even if the effects can be reduced by the insertion of moderating material [30], [31]. Nevertheless, not only the consequences of TRU fuel on safety, but also some technological issues limit the efficiency of SFRs for TRU transmutation in the case of a nuclear phase out. In this case high transmutation rates and long in core residence times are essential. Generally, from the transmutation point of view, conflicting targets always exist, when high transmutation rates are requested:

- a high TRU content in the reactor core is required for efficient transmutation
- no breeding is desired to avoid the built up of new TRU isotopes, thus a fertile free core seems to be optimal
  1. but a very high Pu content is required for a fertile free core
  2. a very high Pu content tends to degrade the Pu vector; this has already been discovered in the CAPRA project [32]
- long cycle time is required for efficient transmutation, since TRUs are burnt only when the reactor operates
- long cycle time requires a small reactivity loss over cycle as a design target for the core, or a core design with high excess reactivity
  1. small reactivity loss over cycle requires breeding of new fissile material, thus fertile material is essential
  2. high excess reactivity has negative safety consequences, since a strong control system is required
- a high burnup is required for an efficient transmutation to reduce the number of recycling steps
- short out of core period and long in core residence time of the TRU fuel is required

The disagreement of the objectives becomes obvious when the requests are combined, but some of the limitations can be smoothen in the MSFR. Fertile free fuel causes a high reactivity loss over the cycle due to the absence of a fertile material, since no breeding of new fissile material occurs. Fortunately, this does not cause any problem in MSR since, in contrast to solid fuelled reactors, the reactivity loss can be compensated by online re-feeding of fissile materials. Thus no excess reactivity is required at all while in solid fuelled fast reactors always a reactivity reserve is needed. The so-called excess reactivity is required to compensate the reactivity loss during the period from one re-fuelling to the next (usually ∼ 9 – 12 months). In fertile free fuel this excess reactivity has to be high to reach an acceptable long cycle time when no breeding takes place to produce fresh fissile material during the cycle like it is done in traditional fast reactor designs. Two choices are possible for the designer. On the one hand it would be possible to reduce the cycle time results in reduced operational performance and prolonged out of operation time of the reactor. Especially in a classical fast reactor, where traditionally the outage time is longer than in LWR, this option is not very attractive. The prolonged outage time in a fast, liquid metal cooled reactor is caused by the more challenging fuel handling operations. On the other hand it is possible to use a high excess reactivity which requires a strong control system with effective single control elements to compensate the excess reactivity at begin of cycle or a very high number of costly and complex control elements which have to be placed in the narrow space above the core. Due to safety reasons, the effectiveness of a single control element is required to be low to limit the reactivity insertion, caused by a faulty movement of a control element. In SFR design, the reduction of the reactivity loss over cycle is one of the main design criteria for the development of modern, well balanced fast reactor designs. It allows limiting the effectiveness of single control rods and thus reduces the possible accident initiator of unprotected transient over power events. This fact leads directly to the safety of the fast reactor system itself. On the one hand, the amount of minor actinides in homogeneous mode in a classical solid fuelled fast reactor core has to be limited, since minor actinides reduce the negative Doppler feedback effect, increase the positive coolant feedback effect and the sodium void worth, and decrease the control rod efficiency [30], [31]. Additional problems arise from the core design (helium production) and the fuel cycle considerations – handling of fuel with high thermal power and considerable neutron source [37]. The delayed neutron production is reduced in the case of the absence of fertile materials. On the other hand, MA transmutation efficiency increases with increasing MA content [30], [31]. In contrast, the structure of the feedback effects is completely different in a fluid fuelled reactor like the MSFR. What is called coolant effect in the SFRs, can be transferred to the density effect of the salt in the MSFR while the Doppler effect appears in both kind of reactors in a comparable way. The salt density effect is strongly negative in all cases, due to the coincidence of coolant and fuel in a molten salt reactor. A decrease in the salt density due to a temperature increase coincides with a decrease of the density of the fissile material dissolved in the salt in the control volume. Thus the MSFR is a very stable system with strong feedback effects which inherently tend to stabilize the reactor at the given power level. The problem of the reduced delayed neutron production is known and has been dealt with in any fertile free core and has already been investigated in the CAPRA concept [32]. So, it should be possible in a MSFR, especially since in the MSFR no excess reactivity is required. Thus possible accident initiators will be limited to a faulty operation of the feeding system. This results in a rather slow transient which will be counteracted by the strong negative feedback effect in the MSFR. The long in core residence time can be assured in the MSR, too, since the salt cleanup is to be performed online on a small partial stream of hot fuel. In this case no extra cooling time of fuel assemblies, no transport to the reprocessing, and no solid fuel pellet production is required. Online reprocessing or salt cleanup has to be designed, but losses in the process do ideally not lead to an accumulation of TRUs in the final disposal stream. In MSFR processing losses will mostly lead to an accumulation of lanthanides in the core, since not the TRUs are extracted from the bulk, but the fission products. Additionally, besides the partitioning of the MA from the waste stream, another major hurdle in the industrial implementation of a transmutation cycle is the production of the solid fuel pellets with high MA content. This process can lead to additional losses and the radiation level during the production process is high. The fuel pellets are complicated to handle and even have to be cooled when the TRU content is high. All these problems are eliminated completely in MSRs. The same can be stated for the major parts of the transports, since only transports from the reprocessing of the LWR fuel to the MSFR are need but no transports due to the required multi recycling in SFRs. This reduces one of the most vulnerable points in nuclear reactor operation since the transports are an important condensation point for public protests in Germany.

**Figure 1:**
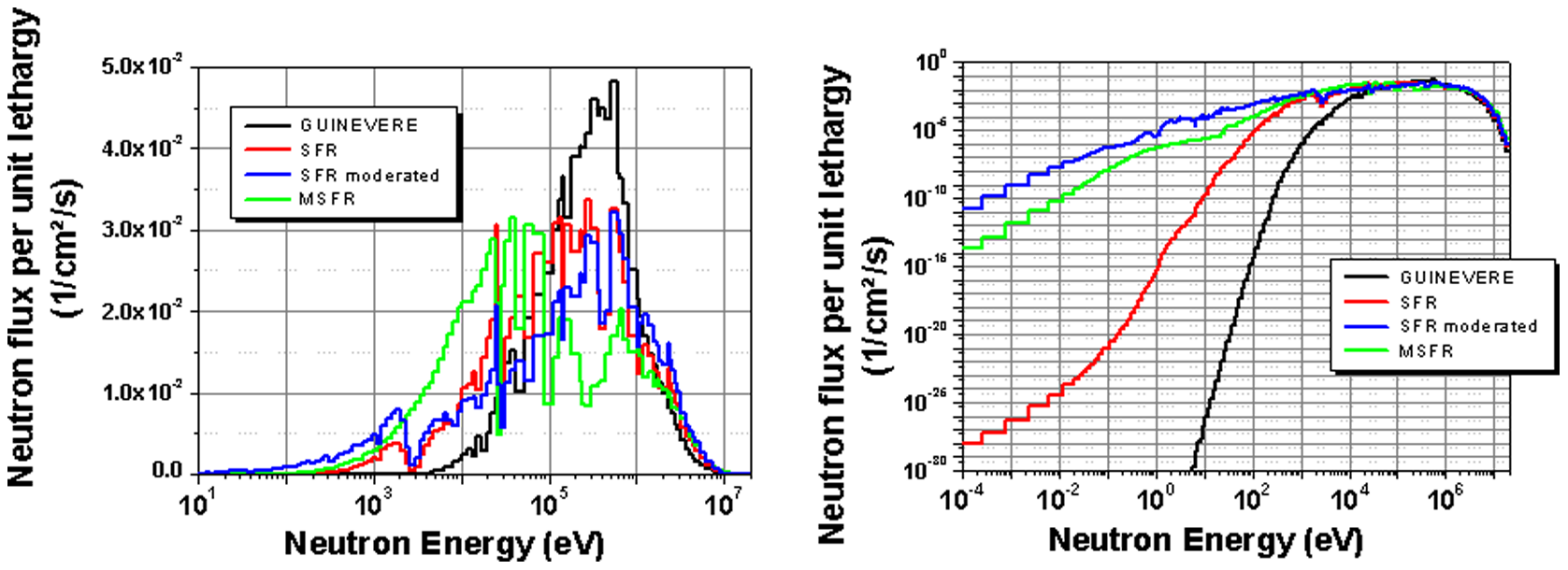
Comparison of the neutron spectrum in different fast reactor systems.

Finally, the neutron spectrum has to be discussed. The neutron spectrum in a MSFR is slightly softer than in other fast reactors; like it is shown in the comparison with an oxide fuelled SFR and the metal fuelled GUINEVERE experiment using a lead matrix (see Figure 1). The reason for the softer spectrum is the high amount of light materials (Li, F) in the core. Nevertheless, it has already been shown that it does not lead to a high accumulation of higher actinides [10]. Additionally, it has been demonstrated that the transmutation efficiency in the slightly softer neutron spectrum of a SFR with fine distributed moderating material (blue curve) is nearly equal or even slightly better [30], [31].

## Method, Code, and Data

**Figure 2:**
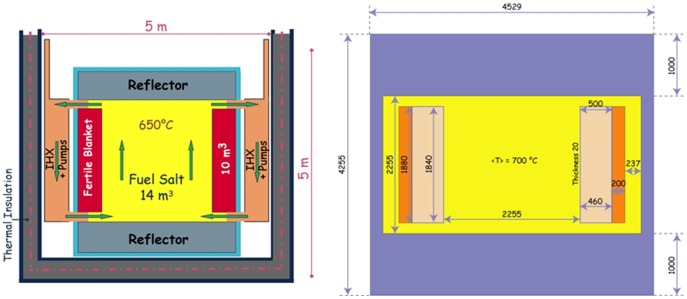
(Left): Simplified to scale vertical scheme of the MSFR system including the core, blanket and fuel heat exchangers (IHX) – (Right): Model of the core as used for the neutronic simulations with the fuel salt (yellow), the fertile salt (pink), the B4C protection (orange) and the reflectors and 20 mm thick walls in Ni-based allow (blue)[12].

In this section the methods of calculation and the used model of the MSFR are described. The transmutation performance of the MSFR is evaluated using advanced computer simulations. The calculations are based on the material configuration, the core dimensions and the boundary conditions of the EVOL benchmark definition. The benchmark calculations performed by several project partners start on a neutronic MSFR benchmark to check the required specific tools and to define together the suitable parameters which will be used further to optimize the MSFR core geometry. The reference MSFR is a 3000 MWth reactor with a fast neutron spectrum and based on the thorium fuel cycle. It may be started either with U-233 or TRU elements as initial fissile load. In a first approach, which has to be refined during the project, the core is a single cylinder (the diameter being equal to the height) where the nuclear reactions occur within the flowing fuel salt inside the cylinder [12], shown in Figure 2. The core is composed of four volumes: the active core, the upper extraction area, the lower injection area, and the out of core area with the heat exchanger and the pumps.

Optimization studies have been performed prior to the beginning of the EVOL project, relying on neutronic considerations (feedback coefficients and breeding capacities), material damages and heat evacuation efficiency, and resulting in a MSFR configuration with a total fuel salt volume of 18 m^3^. Half of the salt is located in the core and half in the external circuits explained above. The salt's thermal hydraulic behavior is closely coupled to its neutronic behavior, because the salt's circulating time (∼4s) and the lifetime of the precursors of delayed neutrons (∼10s) are of the same order of magnitude [12].

The salt configuration consists of 77.5% LiF with ThF_4_-(Pu-MA)F_3_ in the core and with pure ThF_4_ in the blanket. The overall fuel salt volume is 18 m^3^. It contains 30619 kg Th, and 12661 kg TRU following the TRU vector given in Table 1 [13].

**Table 1:** Proportions of transuranic nuclei in UOX fuel after one use in PWR without multi-recycling (burnup of 60 GWd/tHM) and after five years of storage.

Np 237	6.30%
Pu 238	2.70%
Pu 239	45.90%
Pu 240	21.50%
Pu 241	10.70%
Pu 242	6.70%
Am 241	3.40%
Am 243	1.90%
Cm 244	0.80%
Cm 245	0.10%

**Figure 3:**
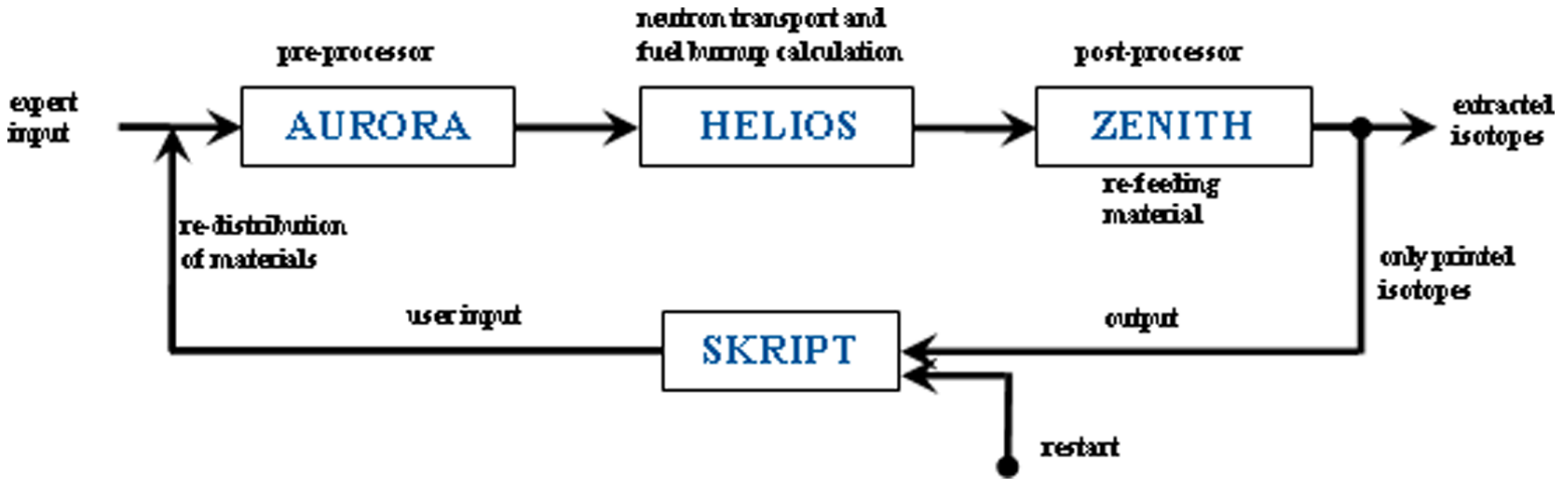
Description of the calculation cycle for the simulation of a MSR.

For the simulations, the HELIOS 1.10 code system with the internal 47 energy group library is used [23]. The code is a 2D spectral code with wide unstructured mesh capabilities and a transport solver, based on the collision probability method [22]. It is written for the calculation of solid structure fuel assemblies, thus the possibility of online refueling and online reprocessing is not foreseen. To deal with these very special features of molten salt reactors a python script has been developed. The script is based on the special features of HELIOS. All important information, which is not changed during the whole reactor operation, is stored in an expert input. The changing material configuration is given in the user input. Both inputs are merged in the pre-processor AURORA, which creates the complete input for the HELIOS calculation run for the determination of the neutron flux distribution and the burnup of the materials for a defined burnup period. The results are evaluated in the post-processor ZENITH. Here it can be decided which isotopes will be fed back into the next user input which is created by the script (see Figure 3) and thus into the reactor itself. Theoretically, it is possible precisely to simulate a molten salt reactor by using small time steps in this calculation loop. In a real MSR two different time scales for the salt cleanup are planned, due to the different extraction methods for the fission products. On the one hand, the helium bubbling, which has a halving time of ∼ 30s for gaseous fission products, is used. On the other hand the online chemical salt cleanup which takes 450 days to have a throughput of 100% of the fuel salt volume. For the required long time investigation an approximation is used, only the second process is simulated and all fission products are extracted after an operation time of 450 days. The salt cleanup is established inside the post-processor, only the isotopes which remain in the salt are forwarded to the script. These isotopes are remaining the reactor for the next burnup step. The isotopes or a share of the isotopes which will be dropped are representing the isotope extraction in the salt processing system. Additionally, a defined amount of material with a given isotope vector, e. g. refill of thorium to the initial amount at each cycle and/or refill of a certain amount of fissile material, can be added to the individual isotopes and will be rewritten by the script into the new user input.

Due to the characteristics of the HELIOS code, developed for solid fuelled systems, some approximations have to be accepted. There is no fuel salt movement in the 2D system, thus an undesired burnup distribution arises during the 450 day cycle. The materials are only re-distributed when a new user input is written in each cycle over the script. The effect could be reduced by reducing the cycle time and a re-feed of a part of the fission products. HELIOS is a LWR code and thus a LWR spectrum is used for the weighting of the 47 group master library. Nevertheless, comparisons between HELIOS-2 and SERPENT on the isotope accumulation during the burnup in SFR have shown a good agreement for the major isotopes [36]. The comparison of the calculated neutron spectra of HELIOS has been compared to different codes during the EVOL benchmark. The approximations for the simulation of the online reprocessing and feeding as well as the use of HELIOS 1.10 with the reduced 47 group library seem to be adequate for the approximation level required for this kind of long term study. Additional approximations on geometry and modeling given by the used benchmark have to be mentioned.

## Different Operational Options for the MSFR

A comparison of different operational options with the use of different core ingredients is given in this chapter, below. In all cases at the beginning of the operation the system is fed with the identical TRU vector to reach criticality. During the whole operation time TRUs are re-fed, but the TRU amounts are different. Additionally, the fertile part in the salt in the core is varied. One system is based on thorium as fertile material, another is based on depleted uranium as fertile material, and the third system is fertile free. In the systems with fertile material, the fertile component is refilled at begin of each cycle. In all cases the bred fissile material from the blanket is taken out of the system at the end of each cycle. In all systems is Th-232 used as fertile material in the blanket – real breeding of fissile material (U-233) takes place. In each case the reactor is operated for 40 cycles with an averaged cycle length which strongly depends on the heavy metal load

**Figure 4:**
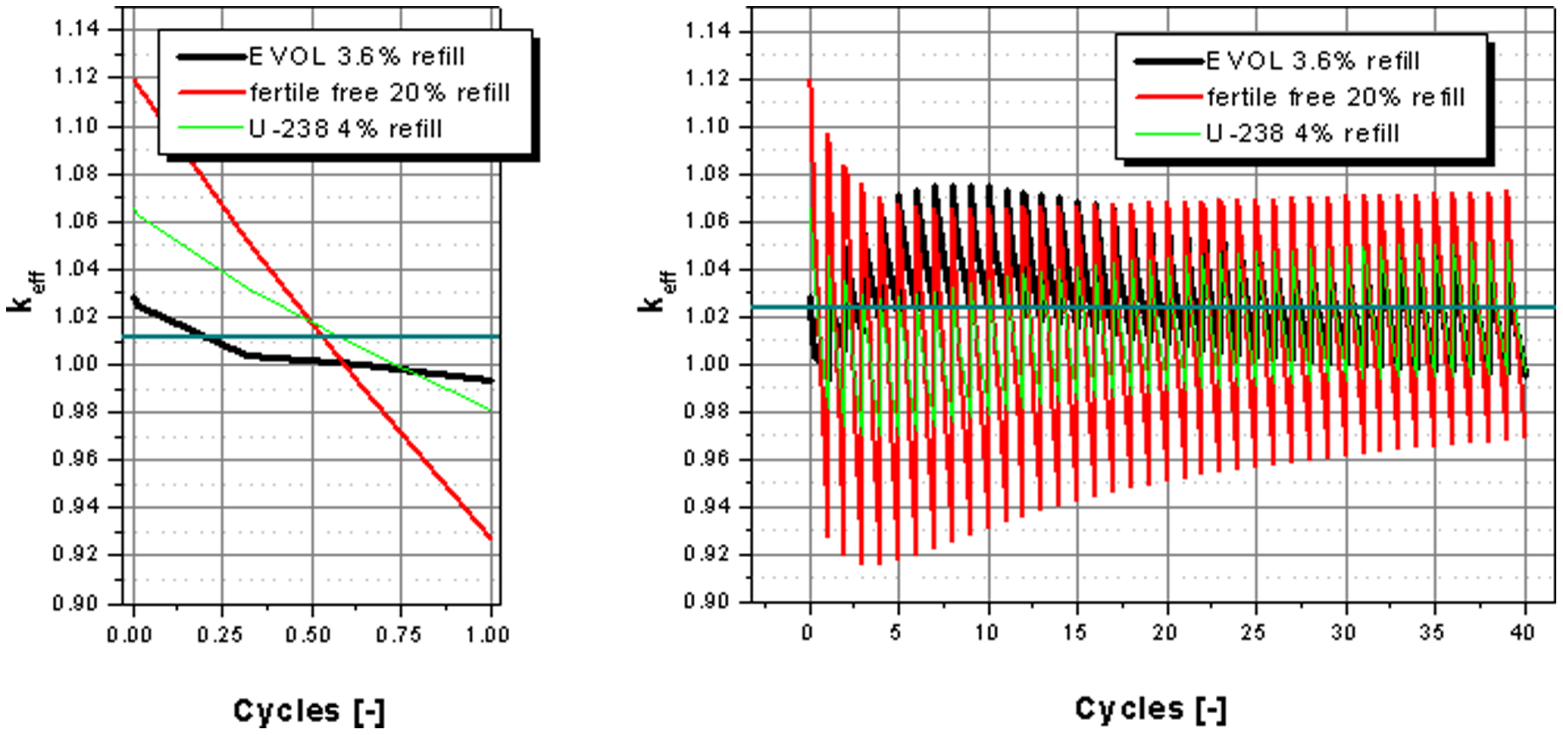
Comparison of the k_eff_ evolution over the first cycle (left) and over the operational period of 40 cycles (right).

1. 40 • ∼443 days ≙ 17720 days or ∼ 48.5 years for the Th-232 case,
2. 40 • ∼62 days ≙ 2480 days or ∼ 6.8 years for the fertile free case, and
3. 40 • 513 days ≙ 20520 days or ∼ 56.2 years for the U-238 case

to achieve the given burnup specified per ton of heavy metal. The k_eff_ and in core inventories of the major TRU isotopes over burnup will be discussed. Figure 4 shows the k_eff_ evolution over the burnup for the period of 40 cycles to reach the specified final burnup. The initial burnup step for all three cases is configured that the average k_eff_ is 1.0112 over the cycle. This value is taken from the critical configuration of the EVOL benchmark definition and is based on the criticality of the 3D system given in the definition [13]. In all three cases, the criticality drops during the cycles, on the one hand due to the burning of fissile material, on the other hand due to the accumulation of fission products (see Figure 4 left). This behavior is a consequence of the approximation of the refill only at the beginning of a cycle and on the approximation of a batch salt clean-up process. Both processes refill and clean-up, would be continuous in a real molten salt reactor. The criticality loss per cycle, caused by the approximation, significantly depends on the breeding capabilities of the fertile component of the fuel. Thus the criticality loss per approximated cycle is much stronger for the fertile free case caused by the small initial amount of fissile material and the absence of breeding processes. The slight swing in the k_eff_ curves over 40 cycles is a result of another approximation, the constant re-feed of TRU at the begin of each cycle.

**Figure 5:**
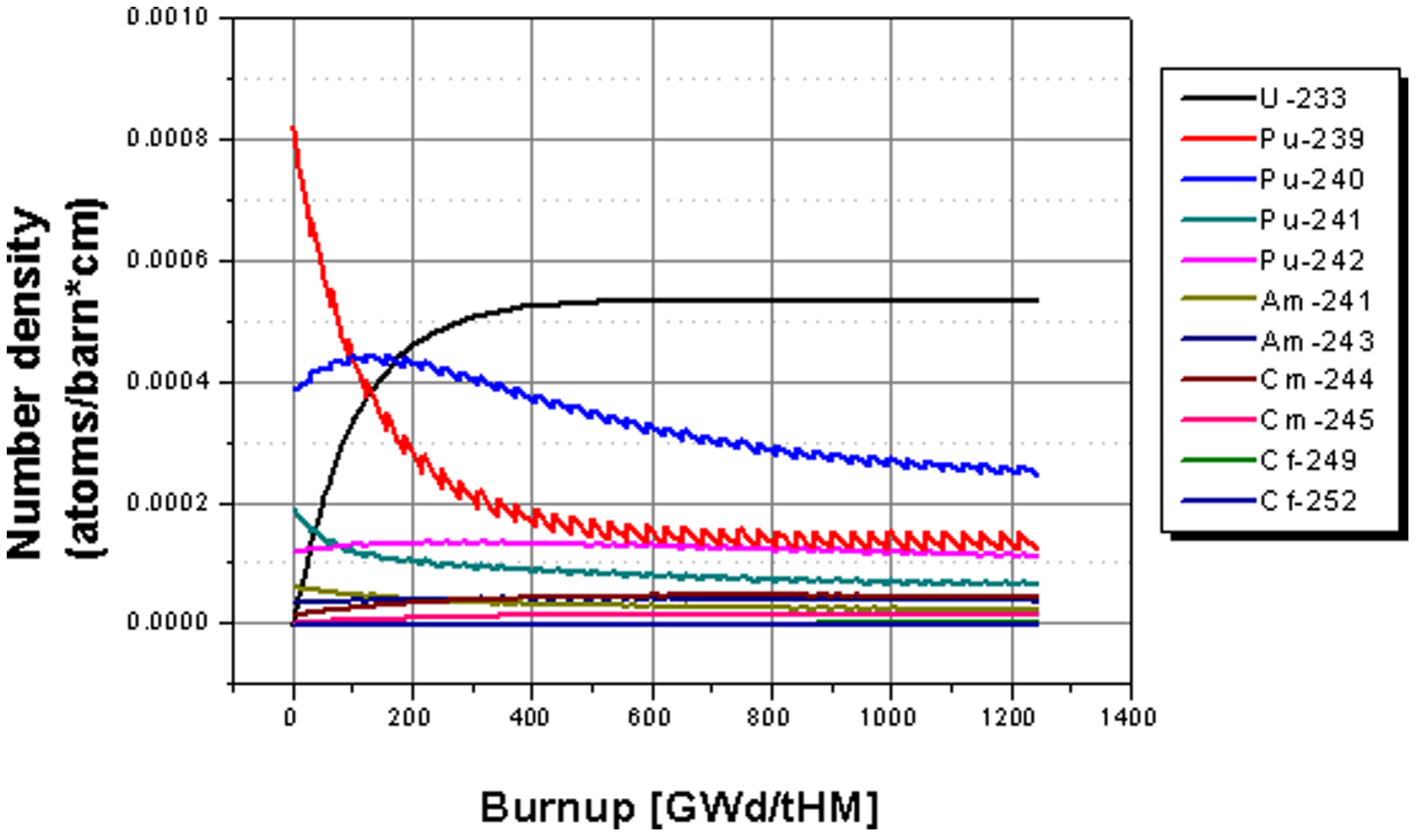
Evolution of the isotope inventory for the case based on Th-232 fertile material.

In the following, the evolution of different important isotopes over 40 cycles is analyzed for the three different cases. The evolution of the isotopic content in the core of the EVOL benchmark system based on the Th-232 fertile system with 3.6% of the initial load re-filled at begin of each cycle is given in Figure 5. The most interesting changes during the operation time are the built up of the U-233 due to breeding processes form Th-232 via a neutron capture to Th-233 and β decays to Pa-233 and then to U-233. The U-233 concentration reaches an asymptotic value roughly after the half of the operation time. The Pu-239 is reduced from the initial value roughly by more than 75%. The appearing steps in the time evolution are caused by the re-feeding of TRU material at the beginning of each cycle for burning. The other fissile Pu isotope Pu-241 is reduced rapidly from the begin of life (BOL) configuration, too, and it reaches an asymptotic value already after short time. The material which is added at each cycle is than burnt during the cycle. The Pu-240 content rises slightly at the beginning of operation to a maximum level at ∼180 GWd/tHM and reduces than to a nearly asymptotic value at the end of operation. The other even Pu isotope Pu-242 is nearly constant over the complete operational period, thus at least the added material is burnt. The americium isotopes Am-241 and Am-243 end with an asymptotic level. The amount of Am-241 is slightly reduced from amount at BOL, while the Am-243 amount rises to reach the asymptotic level. Cm-244 and Cm-245 amounts rise slightly over the operational period, but both isotopes approach an asymptotic level, too. Only marginal amounts of californium are produced during the lifetime of nearly 50 years.

**Figure 6:**
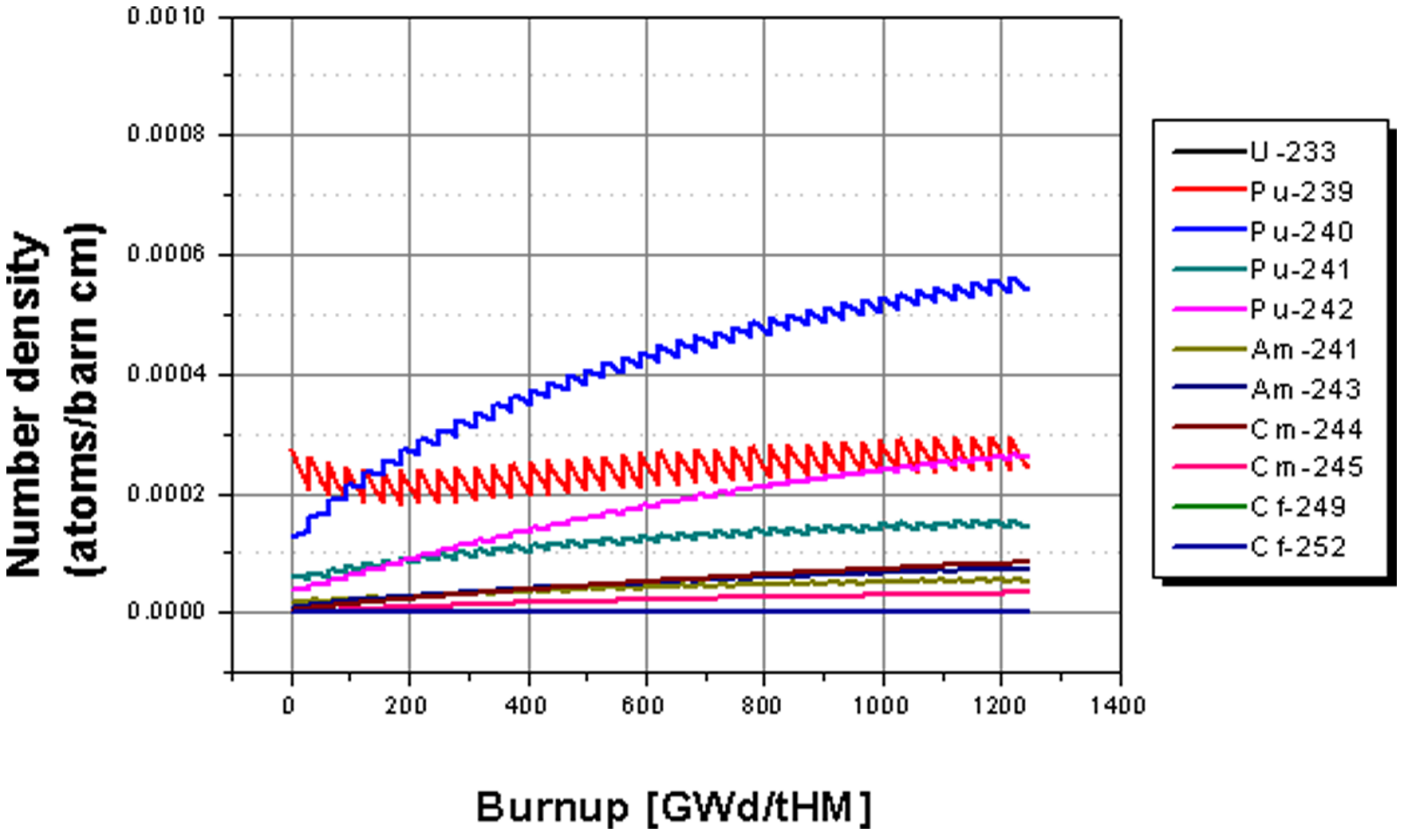
Evolution of the isotope inventory for the case of a fertile free core.

The 40 cycle evolution of the isotopic content in the core of the fertile free system with 20% of the initial load filled at begin of each cycle is given in Figure 6. Already a first glance indicates some important differences for the fertile free system compared with the thorium based system. There is no significant built up of new fissile material due to breeding during operation, thus, on the one hand, significantly more TRUs have to be fed into the system. On the other hand, the initial load is much lower, since there is no fertile material, which absorbs heavily neutrons. The amount of fissile isotopes Pu-239 and Pu-241 is at EOL slightly higher than the initial value, but most of the re-fed material is burnt during the operation. The final inventory of Pu-239 is more than 60% higher, and the Pu-241 content is even more than two times higher compared to the Th-232 based system. The Pu-240, as well as the Pu-242 content, rises strongly over the whole operation time, thus the even isotopes are accumulated and only a small part is burnt. The observed americium and curium isotopes are accumulated in the core, too. The same can be observed for the californium isotopes which accumulate to a final value which is ∼5 times higher than for the thorium based case.

The differences between the two cases can be explained with the lack of fertile material. The required fissile content is slightly lower in the starting configuration of the fertile free system, since the loss of neutrons due to absorption in the fertile material does not exist. In contrast to the thorium based case where the fertile material absorbs neutrons with the result that a significant amount of fissile material is replaced by the bred U-233 over time. To compensate for the absence of significant breeding in the fertile free system, the amount of fissile material which has to be added in each step is significantly higher. Due to the comparably high plutonium concentration in the core a competition between fission and capture events takes place in the fertile free system. Even in the very efficient fissile materials, like the odd plutonium isotopes, the capture leads to breeding of higher even isotopes. Then these isotopes have to undergo more capture events to create new fissile material, since the even isotopes have a significantly lower fission probability even in fast reactors. This processes and effects have already been discovered during the cycle analysis of the CAPRA project [33]. However, from the point of view of a short operation time of a future transmutation facility like it would be desirable in the case of a nuclear phase out, the fertile free option would be significantly more attractive. One of the most interesting points is, that the observed period in this case is only five years, thus the reactor could be operated significantly longer in the fertile free mode until the assumed lifetime of 50 years for the EVOL like system is reached. Unfortunately, the fertile free configuration cannot be one by one applied to the EVOL benchmark configuration due to thermodynamic and chemical boundary conditions like the solubility of the transuranium elements in the carrier salt, the requested melting temperature of the specific salt composition, and the specific configuration required for a successful salt clean-up system. An adoption of the salt composition would be required for an operable configuration of a fertile free system.

**Figure 7:**
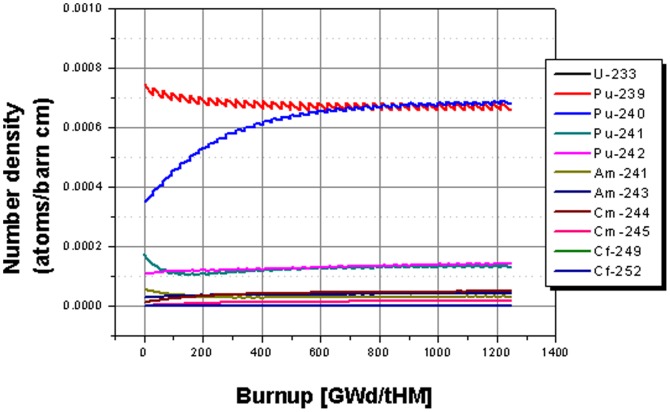
Evolution of the isotope inventory for the case based on U-238 fertile material.

In the following, the use of U-238 as fertile material is investigated, since this configuration could be formed based on the EVOL design with minor changes. The newest salt composition in the EVOL project has already been partly shifted to a composition consisting of a mix of LiF with Uranium and Thorium fluoride [34]. The evolution of the isotopic content in the core for the case of the use of U-238 as fertile material over 40 cycles is given in Figure 7. Due to the use of U-238 as fertile material, the Pu-239 content does not change by a significant amount. The burnt Pu-239 is nearly one by one replaced by new bred Pu-239 from U-238 via a neutron capture to U-239 and then via β-decays to Np-239 and forward to Pu-239. The fissile material is mainly configured by Pu-239 and Pu-241 and there is no replacement to another fissile material like in the case for the thorium fertile material. The initial Pu-241 content is slightly reduced and reaches at end of life almost the steady state value. The concentration of Pu-240 rises over most of the operational period and reaches about end of life (EOL) almost an asymptotic value, too. Pu-242 stays nearly constant over the whole operation. The same can be observed for the americium and the curium content. Only the californium content rises.

The comparison of the three cases shows, that the reduction of the odd plutonium is most efficient in the view of the plutonium content inside the reactor for the case of thorium use as a fertile material, since a major part of the fissile material is replaced by U-233. Additionally, the amount of Pu-240 in the core is the lowest for the case of thorium use. In both other cases the Pu-240 inventory in the core rises with the stronger increase in the fertile free case. The Pu-242 inventory is the lowest for the case with U-238 fertile and the case with thorium fertile. The content is almost constant in both cases, while in the fertile free core the content rises strongly. The higher isotopes (americium, curium and californium) behave mostly constant in both cases with fertile materials, while the in core contents of these elements rise in the case of the fertile free configuration.

The in-core contents of the fissile isotopes (given in Figure 5 to Figure 7) are only a part of the interesting information for the evaluation of the transmutation performance, since different amounts of TRUs are fed into the system in the initial state, as well as in each step during the operational period of 40 cycles. The initial amount of TRU which is put into the system for the start up is given in Table 2 for all three observed cases. The share of burnt material during the operation of 40 cycles with the specific cycle length given Table 2, are given in Table 3 **.** The unloading masses of bred isotopes after 40 cycles normalized to the inserted TRU inventory is given in Table 4 for all three cases.

**Table 2:** Masses which are initially to be put into the core for the three cases (thorium fertile, depleted uranium fertile, fertile free) and whole feeding mass over 40 cycles operational period.

Initial feed			
	Th-232 fertile (EVOL)	U-238 fertile	fertile free
Th (kg)	30619		
U-238 (kg)		30600	
Pu (kg)	11079	9915	1102
Np (kg)	789	706	79
Am (kg)	677	606	67
Cm (kg)	116	104	12
fissload	6.4%	5.8%	0.6%
Sum fissile (kg)	12661	11331	1260
re-feed fissile	3.6%	4.0%	20.0%
re-feed fertile	4%	4.2%	
whole feed			
Th (kg)	81369.99		
U-238 (kg)		80722.8	
Pu (kg)	26634	25382	9701
Np (kg)	1897	1808	691
Am (kg)	1628	1551	593
Cm (kg)	279	266	102
Sum TRU (kg)	30437	29007	11086
Sum HM (kg)	111807	109730	11086
initial cycle time (days)	450	442	45
average cycle time (days)	443	513	62

**Table 3:** Share of burnt material during the operation of 40 cycles for the different cases (thorium fertile, depleted uranium fertile, fertile free).

	burnt material in (%) in 40 cycles		
	Th-232 fertile	U-238 fertile	fertile free
Np-237	90%	95%	91%
Pu-238	54%	64%	21%
Pu-239	94%	69%	90%
Pu-240	73%	31%	52%
Pu-241	86%	73%	76%
Pu-242	62%	54%	26%
Am-241	84%	80%	76%
Am-243	55%	51%	26%
Cm-244	–26%	–35%	–100%
Cm-245	–250%	–268%	–533%
Overall	82%	60%	71%

**Table 4:** Masses of bred material which are unloaded from the core after 40 for the three cases (thorium fertile, depleted uranium fertile, fertile free) normalized by the over all feed of TRU material.

	Unloading masses of bred isotopes per inserted mass of TRU		
	Th-232 fertile (EVOL)	U-238 fertile	fertile free
Np-238	0.0009%	0.0005%	0.0008%
Am-242 m	0.0306%	0.0380%	0.0483%
Cm-242	0.0452%	0.0668%	0.0804%
Cm-243	0.0097%	0.0145%	0.0218%
Cm-246	0.2016%	0.2117%	0.3105%
Cm-247	0.0402%	0.0442%	0.0936%
Cm-248	0.0161%	0.0199%	0.0281%
Bk-249	0.0006%	0.0008%	0.0020%
Cf-249	0.0014%	0.0018%	0.0058%
Cf-250	0.0009%	0.0013%	0.0031%
Cf-251	0.0002%	0.0004%	0.0015%
Cf-252	0.00002%	0.00004%	0.0002%
Sum	0.35%	0.40%	0.60%

The initial amount of TRU, to make the systems critical, is the highest for the case of thorium use as a fertile material, since the absorption in the fertile component Th-232 in the core is stronger than for the fertile component U-238. The lowest initial content of TRU appears for the fertile free core, since no strong absorbing fertile material appears there for the breeding of new fissile material. The overall feed, consisting of the initial feed and the feed over the 40 observed cycles, is given in the lower part of Table 2 (whole feed). The overall feed of TRUs as well as of heavy metal is in the same range for U-238 case and the Th-232 case. The initial feeds are slightly higher for the Th-232 case, but the re-feeds during operations are slightly higher for the U-238 case. Finally, both options add up to comparable values. The overall TRU loading in the fertile free case is by almost a factor of 3 lower in the fertile free case, but this has to be seen in correlation with the significantly reduced cycle time by a factor of ten (given in the last line of Table 2).

The evaluation of the transmutation efficiency – what has really been burnt - for the three different cases is given in Table 3. A detailed comparison shows that all inserted materials are burnt more efficiently when thorium is used as fertile material. The only exceptions are the very low content isotopes Np-237 and Pu-238, which are slightly more efficiently burnt in the case of uranium use as a fertile material. The most important differences appear for the major plutonium isotopes Pu-239, Pu-240, Pu-241, and Pu-242 where the burning is significantly more efficient in the thorium based system, since this isotopes are not re-produced by breeding processes. A view on the major plutonium isotopes identifies the problem of a fertile free system. Only the burning of the fissile isotope Pu-239 shows a good performance in this case and thus the plutonium vector will be worsened strongly during the operation caused by the loss of the fissile material share only. The efficiency of the burning of minor actinides is comparable in the thorium and the uranium based system, only minor differences appear. Only the Am-241 burning is working well in the fertile free system, the curium isotopes are accumulated in a much stronger way than in the systems using a fertile component. The comparison of the overall transmutation efficiency shows an efficient transmutation in the thorium based system with 82% reduction (or 77% if the long lived uranium isotopes are taken into account, too). The transmutation in a uranium based system is less efficient 60% in the considered operation time of nearly 50 years, thus there is no gain in the operation time, but a loss in the efficiency. The use of fertile free fuel seems to be an interesting opportunity, since the transmutation efficiency is acceptable (71%) and the cycle time is strongly reduced. However a method has to be found to deal with the degradation of the plutonium vector and with the curium accumulation in the core during operation which requires a significant increase of the loading of fissile material during the operation time. Additionally, a fertile free system would not be possible based on used the salt configuration of EVOL. The plutonium solubility would not be high enough in this carrier salt.

The unloading masses of bred higher isotopes per inserted mass of TRU for the three different cases are given in Table 4. Here, the tendency of the transmutation efficiency for inserted TRUs is confirmed. The unloading masses of bred isotopes normalized on the inserted TRU amounts are lowest for the thorium case, followed by the uranium case. The highest amount of higher isotopes is bred in the fertile free case. The reason for these differences is easy to explain. The breeding process for these higher actinides starts from the plutonium. The plutonium share in the fissile part is lowest in the thorium case, since a major share is provided by the U-233 bred during operation. The plutonium share is higher in the uranium case, since during operation new plutonium is bred from U-238. Thus more plutonium is available not only for fission processes but also for absorption processes. This effect is even stronger in the fertile free case, where higher plutonium isotopes are accumulated during operation.

An evaluation of the transmutation performance in comparison with the CAPRA concept [32] based on solid fuel is given in Table 5. It is obvious that the pure Plutonium burning is more efficient in the CAPRA concept than in the MSFR configurations using fertile material. Nevertheless, as soon as the view is not only focused on Plutonium, but on the whole story of burning the transuranium elements the thorium based core configuration is nearly as efficient as the CAPRA concept, since in CAPRA a significant amount of minor actinides is produced. In this concept form the beginning of the transmutation studies the burning of minor actinides has not been in the focus, especially due to the strong effect of minor actinides on the safety parameters of sodium cooled fast reactors. Additionally, it has to be kept in mind that the studied CAPRA configuration does not result in an equilibrium composition which can be operated under the given boundary conditions in a critical fast reactor [33]. Thus the achieved high plutonium burning rate is somewhat artificial. The use of the fertile free configuration would open a new dimension for the transmutation since the transmutation would be significantly more efficient, but a special salt configuration would be required to achieve a high enough plutonium solubility.

**Table 5:** Comparison of the efficiency in burning of Plutonium and transuranium isotopes (Pu, Np, Am, Cm) between the MSFR and the 4/94 CAPRA oxide reference core.

	kg/TW_th_h	kg/Tw_e_h*
Th-232 fertile Pu burnt	17.6	44.1
U-238 fertile Pu burnt	9.4	23.6
fertile free core Pu burnt	39.9	99.7
**CAPRA Pu burnt**	**29.7**	**74.2**
Th-232 fertile TRU burnt	23.8	59.4
U-238 fertile TRU burnt	11.9	29.7
fertile free core TRU burnt	44.6	111.5
**CAPRA TRU burnt**	**25.8**	**64.5**

* η = 0.4 is assumed for the conversion between thermal power and electric power.

The value of 44.6 kg/TW_th_h confirms the results of the simulations, since this value can easily be determined by a hand calculation. The energy per fission is ∼200 MeV, or 8.9*10^−18^ kWh. This multiplies to 1.12*10^26^ fissions/TWh, considering Avogadros constant and an average weight of ∼240 g/mol leads to ∼44.8 kg material which has to be fission to produce one TW_th_h.

Based on these positive results for the case of thorium use as a fertile material and on the highest maturity of this proposal given in the EVOL project, the study will be continued for this case, now. The objective is the development of a solution for the so called ‘last transmuter’ problem – what can be done to reduce the TRU inventory of the last reactor core of the transmutation facility when the transmuter has to be shut down after all TRUs have been inserted for transmutation.

**Figure 8:**
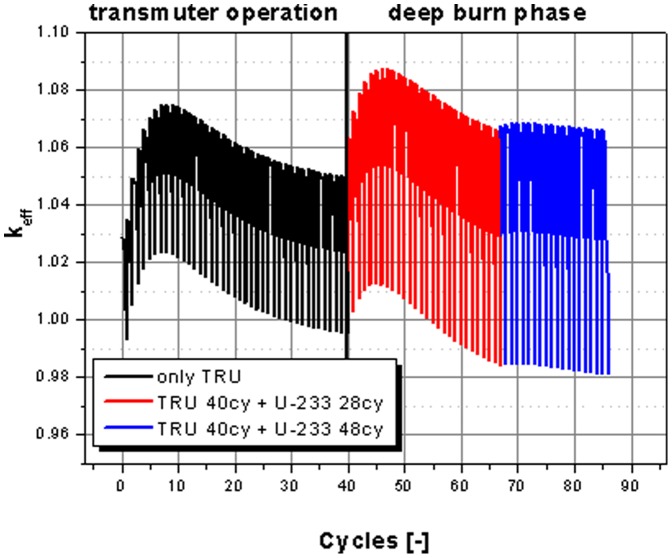
k_eff_ evolution over burnup for the 40 operational cycles (transmuter operation) and the extended operation with U-233 feed (deep burn phase).

Figure 8 shows the operation of the Th-232 based system for 40 cycles using the TRU fuel for the re-fill at each step (black curve), as it has been used before. This period is called transmuter operation. Now, this operational mode is extended in time by 28 cycles (red curve), and the limit case with 20 additional cycles (blue curve). This period is the so-called deep burn phase. For this continuation no more TRUs are fed into the system. Now, the U-233 is used as a fissile material to feed the reactor, which is bred in the LiF-ThF_4_ loaded blanket of the reactor (see Figure 2) over the 40 cycles. A rough estimation indicates that the bred U-233 of the 40 cycles is enough to keep the reactor critical for ∼ 28 cycles. The limit of operation is the use of all bred material of all cycles by direct re-feeding of the fissile material bred over the whole operational period. This asymptotic approach leads to an overall operation time of 40 cycles using TRUs as a fissile material and over all 48 cycles using the U-233, which is bred in the blanket as new fissile material. These 88 cycles represent an operational period of ∼108 years. The time for the operation with U-233 as a fissile material for deep burning of TRU is strongly dependent on the system design and the dimension of the blanket. Thus the used value is specific for the EVOL benchmark design and can be used as an optimization parameter. The dimension of the blanket can be optimized, but the amount of fissile material bred is limited by the leakage of neutrons from the core region. Thus there is neutron physical limit for the maximal amount of U-233 which can be bred.

**Figure 9:**
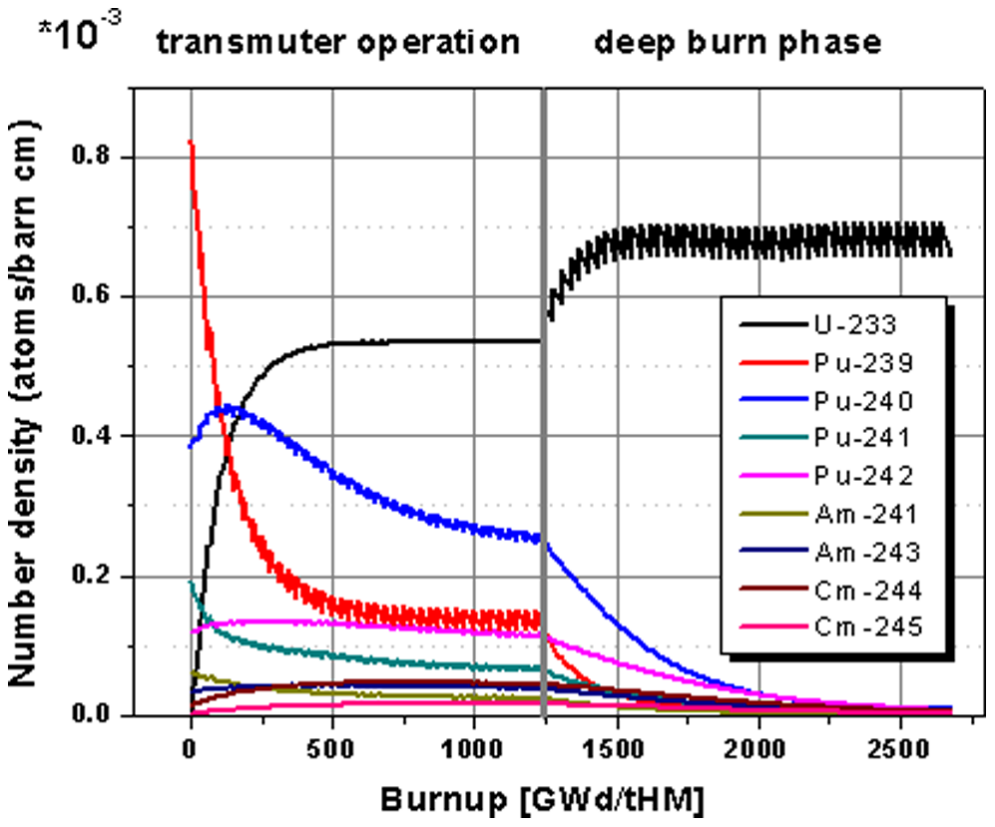
Evolution of the isotope inventory for the case based on Th-232 fertile material with TRU feed in the transmuter operation and the deep burn phase using the U-233 bred in the blanket.

The evolution of the isotopic content in the core for the case of the use of thorium as fertile material over the extended operational period of 88 cycles is given in Figure 9. It is easy to distinguish the two different operation modes (transmuter operation and deep burn phase – separated by the grey vertical line). The transmuter operation is based on TRU feed. It can be identified by the steps in the Pu isotope curves for each cycle. The deep burn phase is based on the feed of U-233 as fissile material. In this phase, the steps appear in the U-233 content coinciding with a rapid growth of the U-233 content in the core. The curves for the TRU isotopes are smooth in this second part, since TRU isotopes are only burnt and not fed into the core anymore. It is obvious from Figure 9, that the deep burn phase has the potential to significantly reduce the TRU leftover in the core at the end of the transmuter operation. In the beginning of the deep burn phase the content of the odd, fissile isotopes Pu-239 and Pu-241 decreases rapidly. This period is followed by the burning of the even plutonium isotopes, for which it is harder to induce fission reactions.

**Figure 10:**
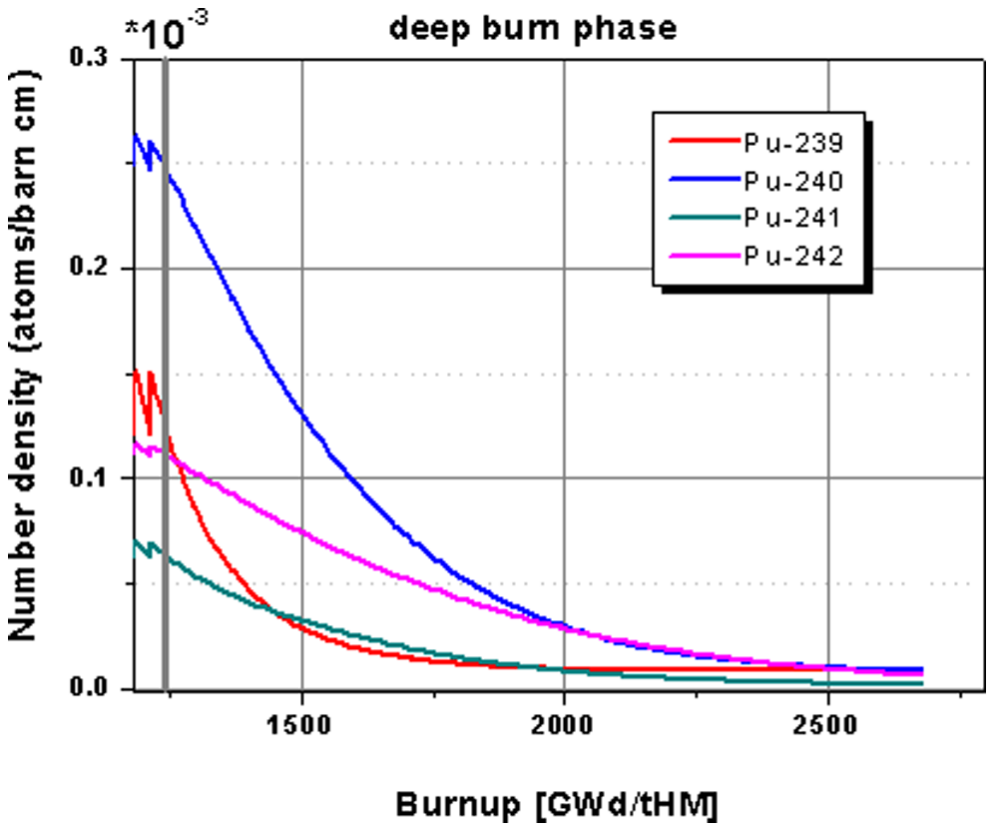
Highlight on the evolution of the plutonium isotope inventory for the continuation in the deep burn phase.

The highlight for the change in the plutonium isotope inventory in the core during the deep burn phase is given in Figure 10. All plutonium isotopes are significantly decreased during the 48 cycle deep burn phase. The major plutonium isotopes Pu-239, Pu-240, and Pu-241 are reduced by nearly 100% from the left over of the transmuter operation based on the case with thorium as a fertile material. Even, the Pu-242 isotope, which is very hard to fission, is reduced by ∼98%.

**Figure 11:**
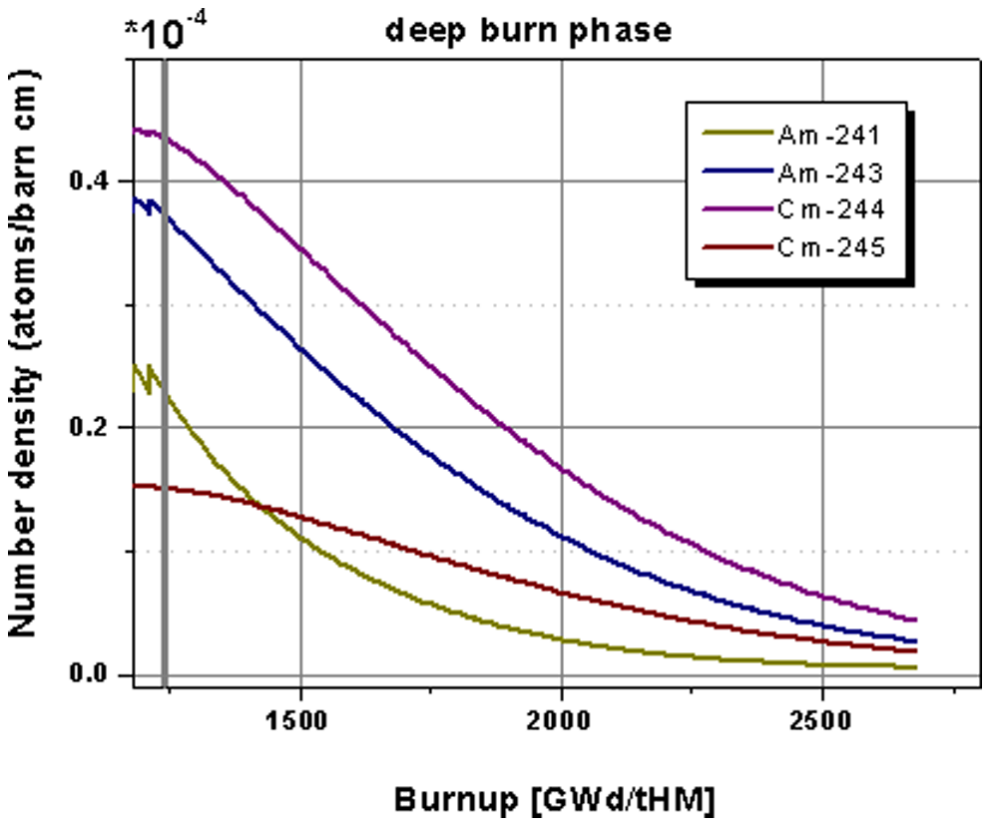
Highlight on the evolution of the minor actinide isotope inventory for the continuation in the deep burn phase.

The highlight on the minor actinide evolution in the core during the deep burn phase is given in Figure 11. All observed minor actinides isotopes undergo a strong reduction in the period where no TRU isotopes are added to the core anymore. The observed americium isotopes are reduced by 99% and 97% and the curium isotopes are reduced by 90 to 60%. Additionally, there is still a strong gradient appearing for Am-243 and the curium isotopes at the end of the observation period. These isotopes are still away from reaching an asymptotic value. Thus an optimized further continuation of the operation, based on U-233 fissile material, would lead to an even stronger reduction of the minor actinide content.

**Figure 12:**
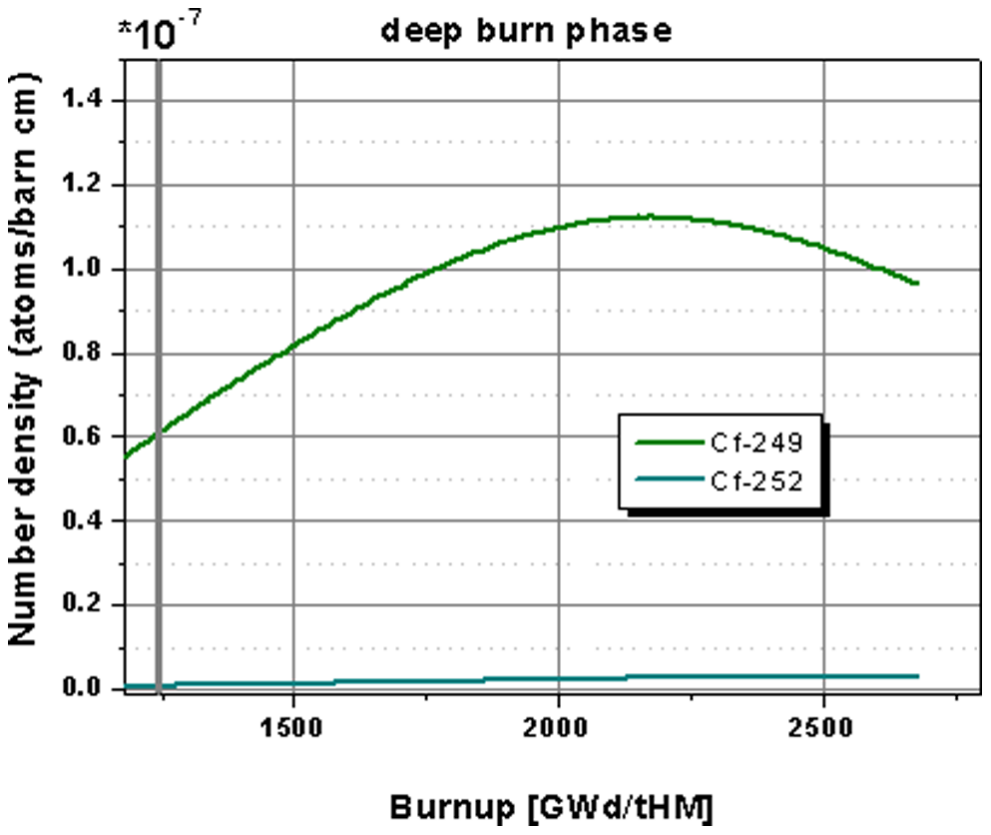
Highlight on the evolution of the californium isotope inventory for the continuation in the deep burn phase.

In Figure 12 the detail of the behavior of the californium isotopes shows a continuous increase especially in the Cf-252 content and a rise to a maximum value followed by a decrease for the Cf-249. Thus a prolonged operation, which would be desirable for an increased burning of minor actinides, improves the burning of Cf-249 too, but it increases the production of Cf-252. Nevertheless, it has to be kept in mind that the absolute values of the californium production are by a factor of 10^4^ lower than the number densities of minor actinides. Additionally, the production of Cf-252 will be reduced when the absolute amount of the Cf-249 decreases, since the breeding chain starts at the Cf-249.

The detailed results for the Th-232 based transmuter operation and the evaluation of results, achieved by the adding the deep burn phase, are given in Table 6. Here, the share of burnt material for transmuter operation period and the transmuter operation plus deep burn phase is presented. It is obvious, that the incineration of all isotopes is significantly increased by the adding of the deep burn phase. All isotopes are strongly reduced during the deep burn phase. The major plutonium isotopes are reduced up to 98% and more. Even the curium isotopes which are produced during the transmuter operation can be significantly reduced in the deep burn phase by nearly 60 to 80% compared to the over all inserted curium. The over all transmutation efficiency is increased from 82% in the transmuter operation to 98% by adding the deep burn phase and could even be further improved by extending the deep burn phase. If the long lived uranium isotopes are taken into account, the transmutation efficiency is only slightly reduced to 77% in the transmuter operation and 93% in the combined operation.

**Table 6:** Isotopic vector of the uranium fuel left after 75 cycles.

	burnt material in %	
	transmuter operation	transmuter operation +deep burn phase
Np 237	90%	97%
Pu 238	54%	82%
Pu 239	94%	100%
Pu 240	73%	99%
Pu 241	86%	100%
Pu 242	62%	98%
Am 241	84%	100%
Am 243	55%	97%
Cm 244	–26%	87%
Cm 245	–250%	58%
Overall	82%	98%

The detailed unloading masses for all observed isotopes after the transmuter operation which are the starting values for the deep burn phase and the unloading masses after the deep burn phase are given in Table 7 to evaluate the changes of the isotopic content during the deep burn phase. In an additional column the reduction achieved due to the adding of the deep burn phase is presented. The overall unloading mass of transuranium elements is reduced significantly (by 89%) compared to the input at the beginning of the deep burn period. Even, when the produced higher even uranium isotopes are taken into account, the reduction is ∼72%. The only isotopes, which increase during the deep burn period, are the ones with atomic masses higher than 247. Overall, less than 7 kg of higher isotopes (isotope mass > 247) are left after the deep burn period where a slight increase appears compared to the 6 kg inserted at the beginning of the deep burn phase. A significant increase appears only in the californium isotopes, but the overall unloading mass of californium isotopes is really limited (∼1.4 kg). Especially for this value it has to be mentioned that the uncertainties can be really high, since the breeding requires a high number of neutron absorption reactions. Additionally, the cross section basis for these high actinides is not of the quality like it is available for the standard materials, often used in nuclear reactors. Thus the absolute values have to be taken with care, but the relative changes in the isotopic contents for the different cases should be trustable.

**Table 7:** Masses which are unloaded from the core after transmuter operation and after the deep burn phase.

	transmuter phase	deep burn phase	
	Th-232 fertile Unloading masses (kg)	U-233 burner Unloading masses (kg)	Reduction (%)
Np-237	192	140	27%
Np-238	0.28	0.23	17%
Pu-238	376	149	60%
Pu-239	857	61	93%
Pu-240	1738	60	97%
Pu-241	443	15	97%
Pu-242	783	45	94%
Am-241	161	4.0	98%
Am-243	263	18.8	93%
Am-242 m	9.3	0.24	97%
Cm-242	14	0.35	97%
Cm-243	3.0	0.09	97%
Cm-244	307	30.7	90%
Cm-245	107	12.9	88%
Cm-246	61	23.5	62%
Cm-247	12	6.88	44%
Cm-248	4.9	5.72	–17%
Bk-249	0.17	0.23	–36%
Cf-249	0.43	0.68	–58%
Cf-250	0.27	0.52	–94%
Cf-251	0.07	0.18	–171%
Cf-252	0.01	0.02	–227%
Sum	5411	575.5	89%

**Figure 13:**
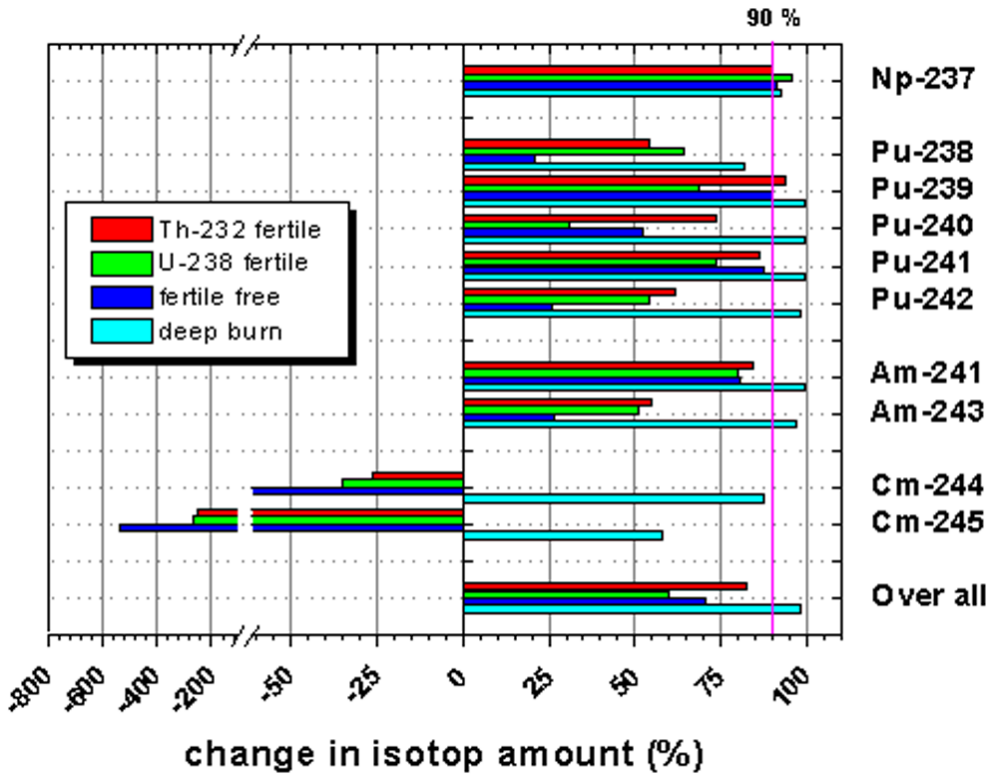
Comparison of the share of burnt material during the transmuter operation of 40 cycles for the different cases (thorium fertile, depleted uranium fertile, fertile free) and by a special add on time, the for deep burn phase of TRU.

Figure 13 visualizes the transmutation efficiency (details are given in Table 3 and Table 6) for the three different cases in transmuter operation and by adding of the deep burn phase. It is obvious, that the incineration of the major plutonium isotopes (Pu-239, Pu-240, and Pu-241), which represent 89% of the plutonium content, is most efficient in the case of the use of thorium as a fertile material and least efficient when U-238 is used as fertile. The transmutation efficiency of all other isotopes is in the same range for the thorium based case and the uranium based case. The use of a fertile free core configuration is in a strong contrast. The burning of the odd, so-called fissile, plutonium isotopes is efficient, but all even isotopes can not be burnt efficiently. Several isotopes are even produced (bars to the left which represent accumulation) in all cases. The closure of the transmuter operation, by adding a second phase, based on the use of U-233 as a fissile material as deep burn phase, increases significantly the transmutation efficiency for all observed isotopes. All TRU isotopes are reduced after this deep burn phase, most of them even by 90% (vertical magenta line) and more. Even curium isotopes which have been produced during the transmuter operation can be significantly reduced in the deep burn phase.

After the operation of a thorium based system a significant amount of uranium is left over, 6.9 tons. The isotopic composition is given in Table 8. It is worth to be discussed what to do with this material. The uranium contains a big share of fissile isotopes ∼74% and could be used as fissile material in nuclear reactors. A transfer into the final disposal seems not to be the aspired solution. Nevertheless, the radiotoxicity and the chemical toxicity of uranium are lower than the one for plutonium and minor actinides. A more attractive solution is the transfer of the fissile material to another country. Due to the elimination of the plutonium no proliferation concerns would arise if the fissile material is given to another country, as long as the proliferation request is fulfilled which defines the maximum share of fissile isotopes to below 20% for U-235 fissile and 12% for U-233 fissile. These requirements could be fulfilled by diluting the remaining uranium composition with U-238 to achieve a share of fissile isotopes which allows the transfer to another country. This could be ideally done by producing e. g. light water reactor fuel which requires a maximum fissile enrichment below 5%.

**Table 8:** Isotopic vector of the uranium fuel left after 88 cycles.

U-232	0.21%
U-233	64.4%
U-234	20.4%
U-235	7.2%
U-236	7.8%
U-237	0.01%

## Conclusions

The molten salt fast reactor seems to be the ideal system for transmutation, if the technological challenges can be solved to an acceptable extent. There are some specific advantages of the liquid fuel based system. Due to the absence of solid fuel no complicated production of TRU based solid fuel is required. This change avoids all drawbacks caused by production technology, losses, and irradiation effects known for solid fuel production. Due to the online salt cleanup the losses of the TRU separation from the bulk of fission products can stay in the system if the processes are properly designed. No public issues like transport of TRU materials, like in multi recycling schemes of solid fuelled reactors, arise. Due to the continuous feeding mode no need for excess reactivity is required and the related safety concerns are eliminated. From the safety point of view, on the one hand the strong negative feedback in the whole operational range due to homogeneous mix of fuel and coolant is very attractive, and the possibility of draining the core opens new features for the decay heat removal. On the other hand a closed safety approach for liquid fuelled reactors in conjunction with the fuel processing has not been developed up to now. This is sure one of the major challenges of the next years if molten salt reactors should become attractive for future application.

Sure, there are several technological challenges to be solved. The main challenge is the stability of the structural materials under high temperature, high neutron irradiation, and exposition to corrosive media. Additionally, a mature design has to be defined for a molten salt reactor. A basic design will be the outcome of the EVOL project. Finally, a step by step approach has to be foreseen to gain operational experience, thus a development has to be started with a small low power experimental machine. Such a machine has to be planned, built, and financed. In the EVOL project it has been shown that the material damage due to irradiation in a MSFR is very high, but there are already some investigations published how to reduce the neutron fluence on the safety related structures [35].

Nevertheless, the MSFR has the described very specific advantages. Thus it is a very attractive alternative, especially in combination with a focus on the phase out of electric power production in nuclear power plants, like it is decided in Germany. The study on the transmutation has shown that the transmutation is the most efficient in a system based on thorium as a fertile material, but a fertile free system could be an interesting alternative due to the very short operation time required. The combination of the use of TRU fuel with a fertile component using thorium in the transmuter operation followed by a deep burn phase using the bred U-233 in the blanket leads to a very efficient transmutation, and thus to a very small left over of TRU materials. Up to more than 90% of all plutonium isotopes is burnt. An overall transmutation efficiency of 97% is reached in a time period of ∼108 years. This reduction values open a completely new view on the possibilities of transmutation, since even in the case of a phase out of nuclear energy production like in Germany, it would be attractive to implement transmutation when it is possible to get rid of more than 95% of the TRUs. Especially, if the option to pass the diluted remaining uranium without proliferation concerns is taken into account. These results are a first estimate to demonstrate the possibilities if a system is designed for the specific German task of rapid burning of TRUs in a limited number of facilities. For a more detailed analysis a mature design of a molten salt reactor system would be required and some of the approximations could be improved in detailed studies. The application of a fertile free system could be promising, if the thermodynamic and chemical limitations can be solved. The combination with the deep burn phase could offer a more efficient transmutation than the thorium based system, but detailed studies are required. Additionally, there is definitely a strong optimization potential available for the number versus the size of the facilities which would be required as well as for the search of an optimal operational period in the burner and in the deep burn mode.

Combining all three aspects of the study leads to the conclusion that MSFRs can be a very attractive transmutation system. Especially, with regard to a nuclear phase out decision or at least a nuclear reactor park which is planned without extensive use of fast reactors for energy production.

## References

[1] MacPherson HG (1985) The Molten Salt Reactor Adventure. Nuclear Science and Engineering, 90, : 374–380.

[2] Renault C, Delpech M (2005) MOST Final Report. March 2005, EURATOM Contract number FIKI-CT-2001-20183.

[3] Mathieu L, Heuer D, Billebaud A, Brissot R, Garzenne C, et al.. (2005) Proposal for a Simplified Thorium Molten Salt Reactor. Proceedings of GLOBAL 2005, Tsukuba, Japan, Oct 9–13, 2005, Paper N 428.

[4] EVOL – Evaluation and Viability of Liquid Fuel Fast Reactor System. Available: http://cordis.europa.eu/search/index.cfm?fuseaction=proj.document&PJ_LANG=EN&PJ_RCN=11669355&pid=5. Accessed 2012 Oct 18.

[5] Renault C, Guérard C (2010) The Molten Salt Reactor (MSR). GIF System Development Progress Status, 4th INPRO-GIF Interface Meeting, Vienna, March 1–3.

[6] Ingatiev V, et al.. (2012) Progress in Development of MOSART Concept with Th Support. ICAPP’12, Chicago, USA June 24–28.

[7] Ingatiev V, Feynberg O, Gnidoi I, Merzlyakov A, Smirnov V, et al.. (2007) Progress in Development of Li, Be, Na/F Molten Salt Actinide Recycler & Transmuter Concept. ICAPP 2007, Nice, France, May 13–18.

[8] Mathieu L, Heuer D, Merle-Lucotte E, Brissot R, Le Brun C, et al (2009) Possible Configurations for the Thorium Molten Salt Reactor and Advantages of the Fast Nonmoderated Version. . Nuclear Science and Engineering. 161: 78–89.

[9] Generation IV International Forum (2009) Annual Reoprt, Available: http://www.gen-4.org/PDFs/GIF-2009-Annual-Report.pdf.Accessed 2012 Oct 18.

[10] Merle-Lucotte E, Heuer D, Allibert M, Brovchenko M, Capellan N, et al.. (2011) Launching the Thorium Fuel Cycle with the Molten Salt Fast Reactor. Proceedings of ICAPP 2011, Nice, France, May 2–5, Paper 11190.

[11] Merle-Lucotte E, Heuer D, et al.. (2009) Optimizing the Burning Efficiency and the Development Capacities of the Molten Salt Fast Reactor. Proceedings Global 2009, Paris, France, September 6–11.

[12] Evaluation and Viability of Liquid Fuel Fast Reactor System EVOL, DELIVERABLE D2.1, Design parameters definition for most stable salt flux, rev 3 30/04/2012.

[13] MOLTEN SALT FAST REACTOR, Reference configuration – revised version, 08.10.2011.

[14] Merk B, Broeders CHM (2008) Auswirkungen von politischen Optionen auf die anfallenden Aktinidenmengen im deutschen Reaktorpark. Atomwirtschaft 6, : 404–412.

[15] Merk B, Broeders CHM (2006) Overview of the Amount of Plutonium Generated against the Background of the Fixed Electricity Amount Regulated by Law in Germany. 9th Information Exchange Meeting.P&T, Nîmes, Sept 25–29.

[16] Salvatore M, et al.. (2004) P&T Potential for Waste Minimization in a Regional Context. 8th IEM on P&T, Las Vegas, USA.

[17] Schwenk-Ferrero A (2013) German Spent Nuclear Fuel Legacy: Characteristics and High-Level Waste Management Issues, Science and Technology of Nuclear Installations, Volume 2013, Article ID 293792, 11 pages. Available: http://dx.doi.org/10.1155/2013/293792. Accessed 2013 Jul 17.

[18] Dreizehntes Gesetz zur Änderung des Atomgesetzes, Veröffentlicht im Bundesgesetzblatt Nr. 43 vom 05.08.2011, Seite 1704 Available: http://www.bundesregierung.de/Content/DE/Artikel/2011/08/2011-08-05-gesetze-energiewende.html?nn=392516#doc126540bodyText1. Accessed 2012 Oct 18.

[19] Gesellschaftliche Implikationen der Partitionierungs- und Transmutationsforschung Available: http://www.acatech.de/de/projekte/laufende-projekte/gesellschaftliche-implikationen-der-partitionierungs-und-transmutationsforschung.html.Accessed 2012 Oct 18.

[20] GIF and Generation-IV, Overview (2012) Available: http://www.gen-4.org/PDFs/GIF_Overview.pdf.Accessed 2012 Oct 18.

[21] Gauché F (2012) Advanced Sodium Technological Reactor for Industrial Demonstration. International Workshopp on Prevention and Mitigation of Severe Accidents in Sodium-cooled Fast Reactors, Tsuruga, Japan, June 11–13.

[22] Villarino EA, Stammler RJJ, Ferri A, Casal JJ (1992) HELIOS: angularly dependent collision probabilities Nuclear Science and Engineering 112.

[23] HELIOS Methods, Studsvik Scandpower Nov. 2003.

[24] Merk B, Stanculescu A, Chellapandi P, Hill R (2013) Progress in fast reactor operation and new trends to increased inherent safety. submitted to PLoS One.

[25] Sauvage JF (2009) PHÉNIX, 35 years of history: the heart of a reactor. CEA, July 2009.

[26] Merk B, Weiβ FP (2011) On the use of a moderation layer to improve the safety behavior in SFRs. Annals of Nuclear Energy 38, 5, 921–929.

[27] Merk B, Kliem S, Fridman E, Weiβ FP (2012) Use of zirconium based moderators to enhance the feedback coefficients in a MOX fuelled SFR. Nuclear Science and Engineering 171 2: 136–149.

[28] Merk B, Weiβ FP (2011) Analysis of the influence of different arrangements for ZrH moderator material on the performance of a SFR core. Annals of Nuclear Energy 38, : 2374–2385.

[29] Merk B (2013) Thermal stability of fine distributed moderating material applied to enhance the feedback effects in SFR cores. invited, to special edition Science and Technology of Nuclear Installations Volume 2013 (2013), Article ID 217548, 11 pages.

[30] Merk B, Weiβ FP (2011) On the Use of Moderating Material to Enhance the Feedback Coefficients in SFR Cores with High Minor Actinide Content. ICAPP ’12, Chicago, USA, June 24–28.

[31] Merk B (2012) Moderating Material to Compensate the Drawback of High Minor Actinide Containing Transmutation Fuel on the Feedback Effects in SFR Cores. invited, to special edition Science and Technology in Nuclear Installations Volume 2013 (2013), Article ID 172518.

[32] Languille A, et al.. (1995) CAPRA core studies: The oxide reference option. Proc. Int. Conf. Evaluation of Emerging Nuclear Fuel Cycle Systems (GLOBAL95), Versailles, France, 1995, ANS.

[33] Wiese H-W (1998) Actinide transmutation properties of thermal and fast fission reactors including multiple recycling. JOURNAL OF ALLOYS AND COMPOUNDS Volume: 271 Pages: 522–529.

[34] Capelli E, Beneš O, Konings RJM (2013) Thermodynamic investigation of the LiF-ThF4-PuF3 system. EVOL WP2 Workshop & EVOL-MARS Meeting, 26-28 June 2013, Grenoble, France.

[35] Merk B, Konheiser J (2013) Shielding Studies on an Advanced Molten Salt Fast Reactor Design. Annals of Nuclear Energy 64(2014), 441–448.

[36] Rachamin R, Wemple C, Fridman E (2013) Neutronic analysis of SFR core with HELIOS-2, SERPENT, and DYN3D codes”, Annals of Nuclear Energy 55, http://dx.doi.org/10.1016/j.anucene.2012.11.030.

[37] Buiron L, Varaine F, Verrier D, Ruah D, Massara S, et al. (2009) Heterogeneous Minor Actinide Transmutation on a UO_2_ blanket and on (U,Pu)O_2_ fuel in a Sodium-cooled Fast Reactor – Assessment of core performances, Global 2009, Paris, France, September 6–11, 2009, Paper 9109.

